# Case of Delirium Tremens Treated with the Extract of Hyosciamus

**Published:** 1849-12

**Authors:** R. A. Richardson

**Affiliations:** Brooklyn, Jackson Co. Mich.


ART. II.—*Case of Delirium Tremens treated with the extract of Hyosciamus.* By. R. A. RICHARDSON, M. D., of Brooklyn, Jackson Co., Mich.

DR. FLINT.

*Sir:*—Allow me to place at your disposal, an account of a case of Delirium Tremens, treated with Hyosciamus. I am not aware that this article has ever been used in the treatment of a case of this kind, although it *may* have been, and if you think the *idea* will be worth anything to the profession, you are at liberty to use this article as you think proper.

Aug. 30th, 3 o'clock, P. M.—Was summoned, in haste, to visit H., aet. about 45, strong plethoric habit, sanguine temperament. Had been in the habit of "taking his bitters," daily, for a number of years; had been in a state of inebriation for three or four weeks previous to the above date, and at the time I saw him, was laboring under an attack of Delirium Tremens. Pulse 120, to 125, *full* and *hard*; vessels of face and neck much distended; tongue not much coated. Took 22 ounces of blood; catharticised freely; left a powder of opium, 4 grains, tart. emt., ¼ grain, to be given every third hour; keep the head cool, and prohibit brandy *entirely*.

31st, 9 o'clock, A. M.—Bleeding seemed to have mitigated the symptoms a short time, but they soon returned; antimony produced some emesis; contents of stomach "bilious;" pulse about the same; on the whole, symptoms quite as bad as yesterday; continued opium and antimony every fourth hour; still prohibit brandy.

Sept. 1st, 9 o'clock, A. M.—Patient slept a *little*, say half an hour during night, but this morning symptoms considerably aggravated, so much so, that I feared the case would terminate fatally. Having used hyosciamus in fevers, with more or less cerebral disturbance, with good effect, and as a *dernier*, resort, I resolved to give it a fair trial in this case. Accordingly, I divided 18 grains of the alcoholic extract into 24 pills, one to be given every hour. Still keep him from his bottle.

2d, 8 o'clock, A. M.—Found H. somewhat better; pulse 112 to 115; expression of countenance more natural; bowels moved twice during night without the use of cathartics, save the laxative properties of the henbane; rested better than night previous, but still raving to considerable extent. Continued the henbane, *one* grain every hour; allowed a little rice water; still keep him from his brandy.

3d, 8 o'clock, A. M.—To my surprise, and great satisfaction, I found H. very much improved; bowels moved once this morning, quite natural; pulse down to 80; rather full, but soft; expression of countenance natural; slept quietly most part of night. Says he is "rather faint here," (laying his hand on the epigastrium) and is quite weak "all over." Continued hyosciamus, 1 grain every 3 hours; allowed rice water and beef tea.

4th, 10 o'clock, A. M.—Found patient quite smart; says he "feels like himself again," though quite weak yet. Omitted medicine, and gave light, but nourishing diet.

5th, 6th and 7th.—Continues convalescent. Took in all, 50 grains of the extract, of full strength; was confident it was a good article, from a fair trial of it previously.

